# The Mechanistic Links between Insulin and Cystic Fibrosis Transmembrane Conductance Regulator (CFTR) Cl^−^ Channel

**DOI:** 10.3390/ijms18081767

**Published:** 2017-08-14

**Authors:** Yoshinori Marunaka

**Affiliations:** 1 Department of Molecular Cell Physiology, Graduate School of Medical Science, Kyoto Prefectural University of Medicine, Kyoto 602-8566, Japan; marunaka@koto.kpu-m.ac.jp; Tel.: +81-75-251-5310; 2 Department of Bio-Ionomics, Graduate School of Medical Science, Kyoto Prefectural University of Medicine, Kyoto 602-8566, Japan; 3 Japan Institute for Food Education and Health, St. Agnes’ University, Kyoto 602-8013, Japan

**Keywords:** CFTR, insulin, epithelial Cl^−^ transport

## Abstract

The cystic fibrosis transmembrane conductance regulator (CFTR) Cl^−^ channel belongs to the ATP-binding cassette (ABC) transporter superfamily and regulates Cl^−^ secretion in epithelial cells for water secretion. Loss-of-function mutations to the CFTR gene cause dehydrated mucus on the apical side of epithelial cells and increase the susceptibility of bacterial infection, especially in the airway and pulmonary tissues. Therefore, research on the molecular properties of CFTR, such as its gating mechanism and subcellular trafficking, have been intensively pursued. Dysregulated CFTR trafficking is one of the major pathological hallmarks in cystic fibrosis (CF) patients bearing missense mutations in the CFTR gene. Hormones that activate cAMP signaling, such as catecholamine, have been found to regulate the intracellular trafficking of CFTR. Insulin is one of the hormones that regulate cAMP production and promote trafficking of transmembrane proteins to the plasma membrane. The functional interactions between insulin and CFTR have not yet been clearly defined. In this review article, I review the roles of CFTR in epithelial cells, its regulatory role in insulin secretion, and a mechanism of CFTR regulation by insulin.

## 1. Introduction

**Figure 1:**
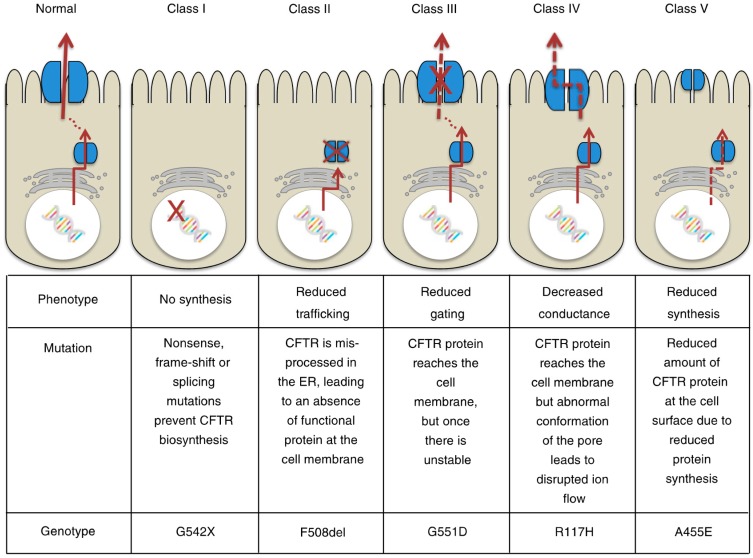
Classification of cystic fibrosis transmembrane conductance regulator (CFTR) mutations. This figure has been published in “Koivula, F.N.; McClenaghan, N.H.; Harper, A.G.; Kelly, C. Islet-intrinsic effects of CFTR mutation. *Diabetologia* **2016**, *59*, 1350–1355 [6]”. This figure is described in this review article under the terms of the Creative Commons Attribution 4.0 International License.

Cystic fibrosis (CF) is the most frequent autosomal recessive lethal disorders in the Caucasian population, and results from loss-of-function mutations in the cystic fibrosis transmembrane conductance regulator (CFTR) gene [1,2,3]. This gene is located on the long arm of chromosome 7 in humans and encodes a polytopic integral membrane protein that functions as a cAMP- and phosphorylation-regulated Cl^−^ channel at the apical surface of secretory epithelia [4,5]. The CFTR Cl^−^ channel is categorized as ABCC7, a member of the ATP-binding cassette (ABC) transporter superfamily [2,3]. The molecular structure and channel gating regulation of the CFTR Cl^−^ channel has been extensively studied. The CFTR Cl^−^ channel is composed of five domains: (1) two membrane-spanning domains (MSD1 and MSD2) form the pore of the channel with a permeability to Cl^−^ and HCO_3_^−^, secreting these ions across the membrane, and each domain is composed of six transmembrane segments (TM1–TM6 and TM7–TM12); (2) two cytosolic nucleotide-binding domains (NBD1 and NBD2), at which ATP is hydrolyzed, regulate channel gating; and (3) a regulatory domain (RD) containing multiple phosphorylation sites controls channel activity [3]. Further, the molecular mechanism causing CFTR Cl^−^ channel dysfunction has been also investigated in depth, and the dysfunction is classified into five types (Classes I−V) (Figure 1) [6]. Dysfunctions of CFTR Cl^−^ channels, categorized as shown in Figure 1 [6], cause infection of bacteria and viruses due to little or lack of water secretion driven by Cl^−^ secretion through the CFTR Cl^−^ channel in epithelial tissues [7] as well as other disorders in non-epithelial tissues, such as pancreatic insulin-secreting β cells [6,8]. In this review article, I review roles of the CFTR Cl^−^ channel in the regulation of epithelial water secretion, regulatory mechanisms of insulin secretion, and the insulin action on the CFTR Cl^−^ channel.

## 2. Roles of CFTR Cl^−^ Channel in Epithelial Cl^−^ Secretion

Transepithelial Cl^−^ secretion from the interstitial space to the apical one is mediated via two steps: (1) the first step is Cl^−^ uptake into the intracellular space via Cl^−^ transporters such as Na^+^-K^+^-2Cl^−^ cotransporter (NKCC) located at the basolateral membrane, and (2) the second step is Cl^−^ release from the intracellular space via CFTR Cl^−^ channels expressed at the apical membrane [9,10,11,12,13,14]. Transepithelial Cl^−^ secretion carries negative charges followed by the movement of cations, such as Na^+^ and K^+^ [15,16,17,18,19,20,21,22,23,24]. The secretion of NaCl and KCl generates osmotic gradients, which produce water secretion [15,16,17,18,19,20,21,22,23,24]. To elevate transepithelial Cl^−^ secretion, at least one of these transporters and channels has to be stimulated [25,26,27,28,29]. On the one hand, functional deficiency of these transporters and/or channels leads to disorder in transepithelial Cl^−^ secretion, which dehydrates the apical space of epithelial tissues resulting in an increase in bacterial and viral infectivity [25,26,27,28,29]. This means that the discovery of factors regulating transepithelial Cl^−^ secretion [9,10,11] is essential to control the amount of apical surface-covering fluid and prevent infection of bacteria and viruses.

Many studies indicate the physiological and pathophysiological roles of NKCC and CFTR Cl^−^ channels in water secretion [30], cell differentiation [31,32,33,34], cell growth [35,36,37,38,39], gene expression, and cell signaling [40,41,42,43,44,45]. More than 2000 mutations with functional deficiencies have been found in the CFTR gene, including the deficiency of intracellular trafficking of the CFTR Cl^−^ channel, leading to a variety of clinical symptoms in CF patients [46,47]. The ΔF508 mutation of CFTR is a deletion of phenylalanine 508 in NBD1, and is the most common cause of CF [46]. The ΔF508 mutation occurs in approximately 85% of CF patients, and presents a deficiency of intracellular translocation to the apical (plasma) membrane due to CFTR misfolding and endoplasmic reticulum (ER) retention [5].

## 3. Action of Insulin in the Kidney and Lung under Conditions with Insulin Resistance

Insulin is a well-known hormone to reduce the level of blood sugar via the stimulation of glucose uptake into muscle cells and adipocytes, etc. by binding to its receptor in the target cells. Insulin receptor, a transmembrane protein with tyrosine kinase activity, is activated via autophosphorylation by insulin binding, which transduces its signal into the intracellular space through a phosphoinositide 3-kinase (PI3K)-Akt-dependent cascade [48]. PI3K-Akt-dependent signals coordinate glucose metabolism [49], ion/glucose transport [48,49,50,51,52,53,54,55,56,57,58], cell growth [49], and cell survival [49]. Insulin stimulates ENaC surface expression in the apical membrane of the distal nephron [57,59,60] and elevates the activity of ENaC expressed in the distal nephron [53], leading to an increase of ENaC-mediated Na^+^ reabsorption in the kidney. It is well known that hypertension is frequently observed in type 2 diabetes mellitus (DM) [61], and this might be due to the hyper-elevation of ENaC-mediated renal Na^+^ reabsorption in hyper-insulinemia. However, insulin resistance is commonly observed in type 2 DM [62]. This means that insulin might not stimulate ENaC-mediated renal Na^+^ reabsorption due to insulin resistance, even under hyper-insulinemia conditions in DM. Aoi et al. reported that the pH of interstitial fluid is lower in type 2 DM than in healthy controls [61]. This lowered interstitial fluid pH causes insulin resistance via the reduction of insulin binding affinity to its receptor [48,63,64,65], while the interstitial fluid pH in the kidney and lung might be higher (normal) owing to a large amount of blood flow compared with the interstitial pH in skeletal muscle cells and adipocytes. This means that hyper-insulinemia would elevate ENaC-mediated Na^+^ reabsorption in the kidney and lung with a normal interstitial fluid pH in type 2 DM patients, who suffer from insulin resistance in skeletal muscles and adipocytes due to the lowered interstitial fluid pH. This would be a reason why hypertension is frequently observed in type 2 DM: i.e., insulin could elevate ENaC-mediated Na^+^ reabsorption in the kidney, overloading body fluid volume even under conditions with insulin resistance, since insulin resistance might not be observed in the kidney due to the normal interstitial fluid pH, unlike the lowered interstitial fluid pH that is found around muscles and adipocytes.

## 4. Roles of the CFTR Cl^−^ Channel in Insulin Secretion

**Figure 2:**
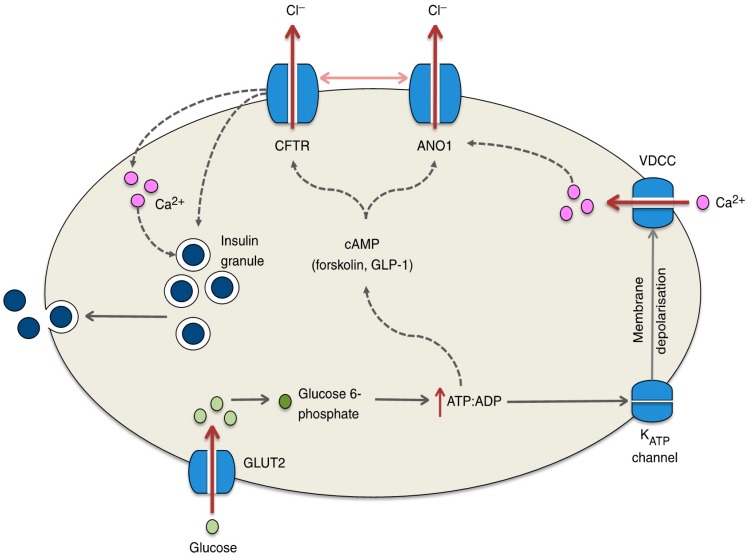
Potential mechanisms by which CFTR regulates insulin secretion from the β cell. Glucose enters the β cell through GLUT2 and is rapidly metabolized to glucose 6-phosphate, ultimately resulting in the generation of ATP, which causes the ATP-sensitive K_ATP_ channel to close. Membrane depolarization and the opening of voltage-dependent Ca^2+^ channels (VDCCs) ensue and calcium fluxes into the cell, resulting in insulin exocytosis. Recent studies have suggested that this process is hampered in the absence of CFTR, which may result from defects in ATP-generated cAMP activation of the CFTR channel. Indeed, pronounced reductions in insulin secretion are observed in response to forskolin- and GLP-1-stimulated increases in the cAMP level. In addition, evidence suggests that CFTR (in conjunction with ANO1) may be involved in the priming of the insulin granule or in the regulation of the calcium flux within the β cell. The regulation of ANO1 by CFTR is denoted by the horizontal arrow between the two channels; dotted lines represent proposed mechanisms yet to be confirmed. This figure has been published in “Koivula, F.N.; McClenaghan, N.H.; Harper, A.G.; Kelly, C. Islet-intrinsic effects of CFTR mutation. *Diabetologia* **2016**, *59*, 1350–1355 [6]”. This figure is described in this review article under the terms of the Creative Commons Attribution 4.0 International License.

It has recently been reported that most patients with CFTR gene mutations exhibit an insufficiency of insulin secretion [66,67], causing DM in CF patients; this is referred to as CF-related diabetes (CFRD) [68]. However, we found little information on the molecular mechanism that causes insufficiency of insulin secretion in CF patients. Edlund et al. [69] reported that: (1) the CFTR Cl^−^ channel has a novel function as a regulator of insulin secretion and exocytosis in pancreatic β cells by contributing to the glucose-induced membrane depolarization, and (2) the CFTR Cl^−^ channel also plays a role in the regulation of ANO1, which participates in glucose-induced membrane depolarization (see Figure 2). The glucose-induced membrane depolarization due to Cl^−^ efflux through CFTR Cl^−^ channels and ANO1 stimulates downstream priming of insulin granules prior to the fusion and release of insulin [69] (Figure 2). Thus, impaired insulin secretion in CF patients would be caused by an insufficiency of membrane depolarization due to the lack of Cl^−^ efflux via CFTR Cl^−^ channels and ANO1. Guo et al. [70] similarly reported that the glucose-induced insulin secretion and membrane depolarization are abolished or reduced by the knockdown or application of CFTR Cl^−^ channel inhibitors such as CFTRinh-172 and glyH-101 in primary mouse pancreatic β cells or RINm5F β cell line. Their study [70] also indicated that glucose-induced insulin secretion and membrane depolarization are significantly diminished in CFTR mutant ΔF508 mice compared with wild-type mice. These observations imply that CFTR Cl^−^ channels play an important role in glucose-induced membrane depolarization, which stimulates insulin secretion in pancreatic β cells via the elevation of the cytosolic Ca^2+^ concentration [Ca^2+^]_c_.

Guo et al. [70] also measured the intracellular Cl^−^ concentration ([Cl^−^]_i_) using *N*-(ethoxycarbonylmethyl)-6-methoxyquinolinium bromide (MQAE), a Cl^−^-sensitive fluorescent dye, which has been established to be useful for measurement of [Cl^−^]_i_ [71,72,73,74]. They [70] reported that the [Cl^−^]_i_ of RINm5F β cell line is about 100 mM under the basal condition, and application of CFTRinh-172 (an inhibitor of the CFTR Cl^−^ channel) increases [Cl^−^]_i_ about 26 mM [70]. This means that the electrochemical potential of Cl^−^ in the intracellular space is larger than that in the extracellular space. The membrane potentials of pancreatic β cells expressing wild-type CFTR Cl^−^ channels are −61~−67 mV [70]. CFTRinh-172 or ΔF508 expression causes the membrane to be more hyperpolarized to −75 mV [70]. Thus, CFTR Cl^−^ channels function as a Cl^−^-permeable, Cl^−^-releasing pathway maintaining the membrane depolarization [70]. Moreover, the expression of loss-of-function-mutated ΔF508 CFTR Cl^−^ channels diminishes the glucose-induced membrane depolarization and elevation of [Ca^2+^]_c_ due to the activation of voltage-dependent Ca^2+^ channels [70]. This results in an insufficiency of insulin secretion. An interesting point is the higher intracellular Cl^−^ electrochemical potential in pancreatic β cells. In general, Cl^−^ uptake into the intracellular space is mediated via active Cl^−^ transporting systems, such as Na^+^-Cl^−^ cotransporter (NCC) and/or NKCC, driven by the Na^+^,K^+^-ATPase-generated Na^+^ chemical potential difference between the intracellular and extracellular spaces: the intracellular Na^+^ chemical potential < the extracellular Na^+^ chemical potential. Therefore, if we could increase the [Cl^−^]_i_ by elevating the NCC- and/or NKCC-mediated Cl^−^ uptake, the insufficiency of insulin secretion would be improved via membrane depolarization due to elevation of Cl^−^ efflux from pancreatic β cells of ΔF508 CFTR-expressing CF patients.

## 5. Roles of the CFTR Cl^−^ Channel in Insulin Action on Glucose Uptake and the Transepithelial Resistance in Epithelial Tissues

Recently, it has been reported that glucose transporter (GLUT) 4 is expressed in normal human primary airway epithelial cells, and that insulin stimulates the GLUT-mediated glucose uptake in airway epithelial cells similar to skeletal muscle cells via the activation of GLUT translocation to the plasma membrane [75]. Molina et al. [75] also reported that insulin increases airway barrier function detected as transepithelial electrical resistance associated with a decrease in paracellular flux of small molecules in normal primary human airway epithelial cells. However, in human airway epithelia expressing ΔF508-CFTR, insulin shows no stimulatory action on glucose uptake, no elevating action on the transepithelial resistance, and no diminishing action on paracellular flux of small molecules [75]. Further, Akt1 and Akt2, which are the most important signaling cascades of insulin, show smaller responses to insulin in ΔF508-CFTR airway cells than that in wild-type CFTR airway cells [75]. These results indicate that the function of CFTR Cl^−^ channels is required for insulin to stimulate glucose uptake, elevate the transepithelial resistance, and diminish the paracellular flux of small molecules in airway epithelial cells.

## 6. Insulin Action on the CFTR Cl^−^ Channel in Epithelial Tissues and Its Molecular Mechanism

**Figure 3:**
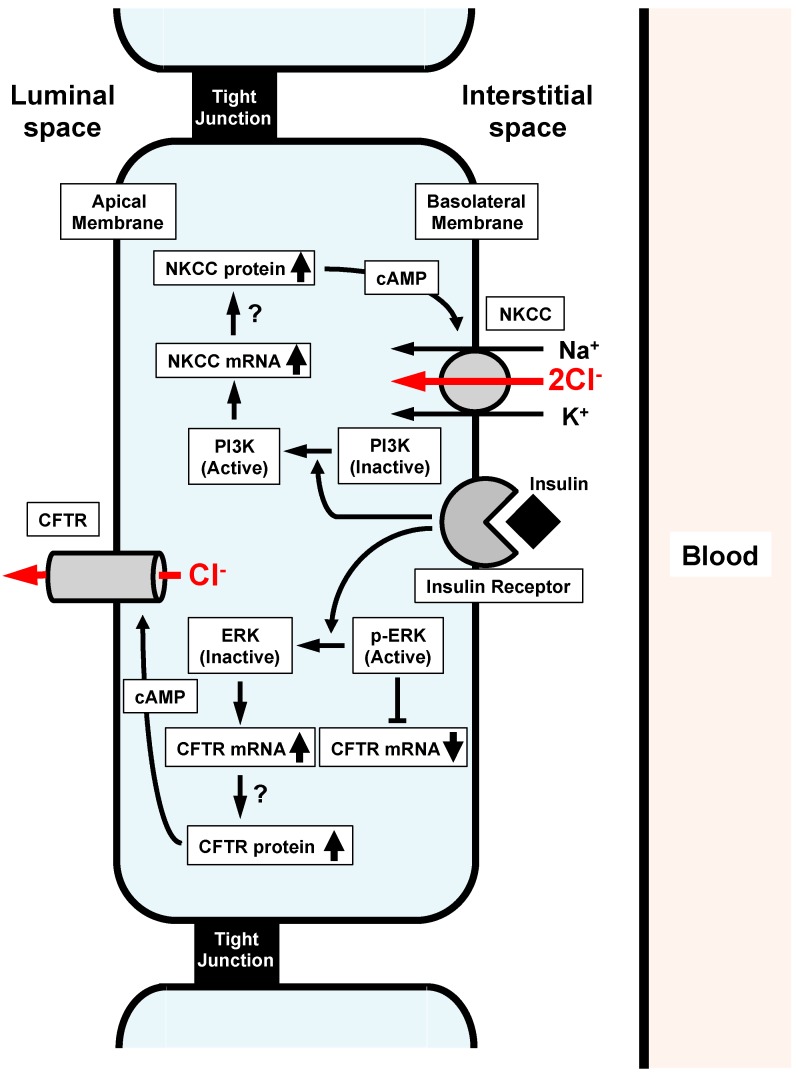
Insulin action on Na^+^-K^+^-2Cl^−^ cotransporter (NKCC) and cystic fibrosis transmembrane regualtor (CFTR) participating in Cl^−^ secretion of epithelial cells. (1) Insulin activates PI3K, resulting in stimulation of NKCC mRNA expression. (2) Insulin inactivates ERK, which suppresses CFTR mRNA expression. Insulin-induced inactivation of ERK releases the suppression of CFTR mRNA expression, leading to the elevation of CFTR mRNA expression. Both the elevation of mRNA expression of NKCC and CFTR induced by insulin might stimulate expression of NKCC and CFTR proteins, which might stay in cytosolic store sites. cAMP respectively stimulates the translocation of insulin-induced NKCC and CFTR proteins staying in cytosolic store sites to the basolateral and apical membranes, leading to a much larger epithelial Cl^−^ secretion associated with a much larger elevation of CFTR activity than that of those under the insulin-untreated condition. Reproduced with allowance of non-profit use of the figure [14].

Another study [14] reported the insulin action on epithelial Cl^−^ secretion. As mentioned above, the epithelial Cl^−^ secretion is mediated by two steps: (1) the Cl^−^ uptake via Cl^−^ transporters such as NKCC across the basolateral membrane, and (2) the Cl^−^ release via CFTR Cl^−^ channels across the apical membrane. Insulin upregulates the mRNA expression of both CFTR Cl^−^ channels and NKCC (Figure 3) [14]. If the insulin-induced upregulation of mRNA expression of CFTR Cl^−^ channels and/or NKCC would elevate the number and/or activity of CFTR Cl^−^ channels at the apical membrane and/or NKCC at the basolateral membrane, epithelial Cl^−^ secretion would increase [13]. However, insulin application alone to epithelial cells has no effect on Cl^−^ secretion or CFTR Cl^−^ channels expressed at the apical membrane [14]. This means that insulin does not increase the number or activity of CFTR Cl^−^ channels at the apical membrane or NKCC at the basolateral membrane, even though insulin upregulates the mRNA expression of both CFTR Cl^−^ channels and NKCC. Interestingly, under the cAMP-stimulated condition, insulin elevates epithelial Cl^−^ secretion and apical CFTR Cl^−^ channel conductance (activity). This observation suggests that: (1) insulin might increase the production of both CFTR Cl^−^ channels and NKCC proteins; (2) these CFTR Cl^−^ channel and NKCC proteins might stay in the cytosol space but would not be translocated to the apical or basolateral membrane, respectively, under conditions without cAMP stimulation; and (3) cAMP respectively stimulates translocation of these CFTR Cl^−^ channel and NKCC proteins from the cytosolic store sites to the apical and basolateral membranes. This study [14] reported further interesting observations that: (1) the insulin-stimulated CFTR mRNA expression is enhanced by an inhibitor of MEK, PD98059; (2) insulin significantly inactivates ERK, which is a negative regulator of CFTR Cl^−^ channel expression; and (3) PD98059 treatment enhances the insulin-induced elevation of cAMP-stimulated Cl^−^ secretion associated with an increase in the apical CFTR Cl^−^ channel conductance (Figure 3).

## 7. Conclusions

CFTR Cl^−^ channels, one of the ABC transporter superfamily, play essential roles in: (1) water secretion in epithelial tissues, by generating Cl^−^ secretion and prevention against infection of bacteria and viruses, and (2) maintenance of the plasma membrane depolarization by functioning as a Cl^−^ efflux pathway, which causes glucose-induced insulin secretion by increasing the [Ca^2+^]_c_ in pancreatic β cells. Insulin may play a role in barrier immunity via the elevation of Cl^−^ secretion due to the stimulation of CFTR Cl^−^ channel expression by inactivating MEK (a negative factor for the expression of CFTR Cl^−^ channels). Insulin also plays an important role in the prevention against infection of bacteria and viruses by both decreasing glucose contents at the apical space of airway epithelial cells and elevating the resistance of the tight junction (barrier function) of airway epithelial cells. Therefore, a deficiency of CFTR Cl^−^ channels causes: (1) infection of bacteria and viruses due to dehydration and high glucose contents at the airway apical surface, associated with a relatively high permeability of the paracellular pathway, and (2) a decrease in insulin secretion due to the diminution of glucose-induced membrane depolarization caused by a lack of Cl^−^ efflux through CFTR Cl^−^ channels at the plasma membrane of pancreatic β cells.

## Abbreviations

ABC	ATP-binding cassette
[Ca^2+^]_c_	Cytosolic Ca^2+^ concentration
CF	Cystic fibrosis
CFRD	CF-related diabetes
CFTR	Cystic fibrosis transmembrane conductance
[Cl^−^]_i_	Intracellular Cl^−^ concentration
ΔF508	Deletion of phenylalanine at position 508
DM	Diabetes mellitus
ENaC	Epithelial Na^+^ channel
GLUT	Glucose transporter
MSD	Membrane-spanning domains
MQAE	*N*-(ethoxycarbonylmethyl)-6-methoxyquinolinium bromide
NBD	Nucleotide binding domains
NCC	Na^+^-Cl^−^ cotransporter
NKCC	Na^+^-K^+^-2Cl^−^ cotransporter
PI3K	Phosphoinositide 3-kinase
RD	Regulatory domain
TM	Transmembrane
VDCCs	Voltage-dependent Ca^2+^ channels
